# DAILY EXPERIENCES OF UPLIFTS, STRESSORS, AND SUBJECTIVE VIEWS ON AGING IN GERMANY

**DOI:** 10.1093/geroni/igad104.1536

**Published:** 2023-12-21

**Authors:** Anna E Kornadt

**Affiliations:** University of Luxembourg, Esch-sur-Alzette, Luxembourg

## Abstract

People’s subjective experience of aging can vary from day to day and is related to other experiences in people’s daily lives. While stressors have been previously investigated as predictors of subjective aging, less research has taken the role of positive events into account. We were thus interested in the relationship of daily stressors and uplifts on different indicators of daily subjective aging in later life. In a preliminary sample of N = 42 German adults aged 52 - 75 years, participants completed up to 14 daily-diary questionnaires on subjective age (single item and multidimensional) and ageist attitudes, daily stressors and uplifts. All variables showed significant within-person variability, with ICCs ranging from .24 (stressors) to .87 (ageist attitudes). Uplifts were related to daily subjective age: On days with more uplifts, people felt younger. Stressors were not related to subjective age and no relation was found with ageist attitudes for uplifts and stressors. Findings will be contextualized in a cross-cultural sample.

